# Influence of angiogenetic factors and matrix metalloproteinases upon tumour progression in non-small-cell lung cancer

**DOI:** 10.1054/bjoc.2001.2137

**Published:** 2001-11

**Authors:** Y Shou, T Hirano, Y Gong, Y Kato, K Yoshida, T Ohira, N Ikeda, C Konaka, Y Ebihara, F Zhao, H Kato

**Affiliations:** Department of Surgery, Tokyo Medical University, 6-7-1 Nishishinjuku, Shinjuku-ku, Tokyo, 160-0023, Japan; Department of Pathology, Tokyo Medical University, 6-7-1 Nishishinjuku, Shinjuku-ku, Tokyo, 160-0023, Japan; Department of Thoracic Surgery, China-Japan Friendship Hospital, 2 Yinghuadonglu, Chaoyangqu, Beijing, 100029, China

**Keywords:** vascular endothelial growth, basic fibroblast growth factor, platelet-derived growth factor, matrix metalloproteinase-2, matrix metalloproteinase-9, non-small-cell lung carcinoma

## Abstract

We attempted to investigate immunohistochemical expression of vascular endothelial growth factor (VEGF), basic fibroblast growth factor (bFGF), platelet-derived growth factor (PD-ECGF), c-erbB-2, matrix metalloproteinase-2 (MMP-2), and MMP-9 using surgical specimens of 119 non-small-cell lung carcinoma (NSCLC) cases and to evaluate the relationship between the expression levels of each molecule and clinicopathological factors or prognosis. VEGF expression levels were significantly associated with the local invasion (*P* = 0.0001), lymph node involvement (pN-factor) (*P* = 0.0019), pathological stage (p-stage) (*P* = 0.0027) and lymphatic permeation (*P* = 0.0389). PD-ECGF expression levels were associated with pN-factor (*P* = 0.0347). MMP-2 expression levels were associated with pN-factor (*P* = 0.004) and lymphatic permeation (*P* = 0.0056). Also, MMP-9 expression levels showed a significant correlation to local invasion (*P* = 0.0012), pN-factor (*P* = 0.0093) and p-stage (*P* = 0.0142). Multivariate analysis showed VEGF to be the most related to local invasion (*P* = 0.0084), and MMP-2 was the only factor with significant independent impact on lymphatic permeation (*P* = 0.0228). Furthermore, log-rank analysis showed significant association with poor survival by VEGF, bFGF, MMP-2 and MMP-9. Especially, combined overexpression of VEGF and MMP-2 revealed poor prognosis, our study might provide a basis for the better evaluation of biological characteristics and a new therapeutic strategy based on chemotherapy. © 2001 Cancer Research Campaign http://www.bjcancer.com


*British Journal of Cancer* (2001) **85**(11), 1706–1712
© 2001 Cancer Research Campaign
doi: 10.1054/ bjoc.2001.2137, available online at http://www.idealibrary.com on IDEAL

http://www.bjcancer.com

### Influence of angiogenetic factors and matrix metalloproteinases upon tumour progression in non-small-cell lung cancer

**Y Shou[1,3], T Hirano[1], Y Gong[1], Y Kato[1,2], K Yoshida[1], T Ohira[1], N Ikeda[1], C Konaka[1], Y Ebihara[2], F Zhao[3] and H Kato[1]**

[1]Department of Surgery, Tokyo Medical University, 6-7-1 Nishishinjuku, Shinjuku-ku, Tokyo 160-0023, Japan; [2]Department of Pathology, Tokyo Medical University, 6-7-1 Nishishinjuku, Shinjuku-ku, Tokyo 160-0023, Japan; [3]Department of Thoracic Surgery, China-Japan Friendship Hospital, 2 Yinghuadonglu, Chaoyangqu, Beijing 100029, China

**Summary** We attempted to investigate immunohistochemical expression of vascular endothelial growth factor (VEGF), basic fibroblast growth factor (bFGF), platelet-derived growth factor (PD-ECGF), c-erbB-2, matrix metalloproteinase-2 (MMP-2), and MMP-9 using surgical specimens of 119 non-small-cell lung carcinoma (NSCLC) cases and to evaluate the relationship between the expression levels of each molecule and clinicopathological factors or prognosis. VEGF expression levels were significantly associated with the local invasion ($P$ = 0.0001), lymph node involvement (pN-factor) ($P$ = 0.0019), pathological stage (p-stage) ($P$ = 0.0027) and lymphatic permeation ($P$ = 0.0389). PD-ECGF expression levels were associated with pN-factor ($P$ = 0.0347). MMP-2 expression levels were associated with pN-factor ($P$ = 0.004) and lymphatic permeation ($P$ = 0.0056). Also, MMP-9 expression levels showed a significant correlation to local invasion ($P$ = 0.0012), pN-factor ($P$ = 0.0093) and p-stage ($P$ = 0.0142). Multivariate analysis showed VEGF to be the most related to local invasion ($P$ = 0.0084), and MMP-2 was the only factor with significant independent impact on lymphatic permeation ($P$ = 0.0228). Furthermore, log-rank analysis showed significant association with poor survival by VEGF, bFGF, MMP-2 and MMP-9. Especially, combined overexpression of VEGF and MMP-2 revealed poor prognosis, our study might provide a basis for the better evaluation of biological characteristics and a new therapeutic strategy based on chemotherapy. © 2001 Cancer Research Campaign http://www.bjcancer.com

**Keywords**: vascular endothelial growth; basic fibroblast growth factor; platelet-derived growth factor; matrix metalloproteinase-2; matrix metalloproteinase-9; non-small-cell lung carcinoma

It is generally accepted that primary lung cancer represents one of the most aggressive solid tumours and its prognosis is still poor despite intensive application of improved therapy. It is not rare for metastatic lesions to already be discovered at the initial diagnosis. Approximately 80% of primary lung cancer is histopathologically diagnosed as non-small-cell lung carcinoma (NSCLC) (el-Torky et al, 1990), and generally surgical treatments are applied to relatively early stage of NSCLC cases. However, distant metastasis occurs in most of these patients within a few years and local recurrence also appears in a few cases. The 5-year survival rate of surgically treated cases with early-stage NSCLC is only 50–60% (Ginsberg and Rubinstein, 1995). This incident suggests that some molecular mechanisms of lung cancer progression might markedly influence the progression of NSCLC. Recently, some evidence that several kinds of molecules reflect distant metastasis and local invasion of cancer cells has been presented (Gasparini, 1996; Stetler-Stevenson et al, 1996).

It has been clarified that angiogenesis is an essential process required for the growth and metastasis of solid tumours (Folkman, 1985). In this context, several growth factors with angiogenic activity in lung cancer have been reported, such as VEGF, PD-ECGF, FGF (Gasparini, 1996). Some investigations showed the vascularization and progress of metastases associated with the expression of VEGF (Volm et al, 1999; Yano et al, 2000). The expression levels of VEGF in stage I NSCLC were significantly different between cases with recurrence (46.5%) and without recurrence (11.5%) (Ohta et al, 1999). Furthermore, the co-expression of VEGF, PD-ECGE and bFGF were significantly associated with lymph node involvement and prognostic information (Volm et al, 1999; O'Byrne et al, 2000).

On the other hand, it was clarified that the degradation of the extracellular matrix and penetration of basement membranes played an important role in tumour invasion and metastasis (Kleiner and Steler-Stevenson, 1999). Several reports showed that the levels of the matrix metalloproteinase (MMP) family are associated with the lysis of basement membranes and with tumour invasion (Stetler-Stevenson et al, 1996). The expression of MMP-2 or MMP-9 confers a worse prognosis in early stage adenocarcinoma of the lung (Kodate et al, 1997; Passlick et al, 2000). However, a few investigators reported that there was no significant correlation between expression of MMP-9 and the prognosis in patients with NSCLC (Fujise et al, 2000). Though the regulatory pathways for these kinds of molecules seem to be complex, recent research showed that a significant proportion of NSCLC tumours co-express MMP-9 and EGFR, and that their co-expression conferred a poor prognosis (Cox et al, 2000).

*Received 4 April 2001*
*Revised 30 August 2001*
*Accepted 31 August 2001*

*Correspondence to:* T Hirano

In our study, we attempted to clarify the relationship between the expression of VEGF, bFGF, PD-ECGF, MMP-2, MMP-9 and c-erbB-2 in NSCLC and clinicopathological characteristics. Furthermore, the relationship between each molecule and survival was evaluated.

### MATERIALS AND METHODS

### Patients and tumour specimens

Surgical materials were obtained from 119 patients with NSCLC lesions resected between May 1994 and December 1995 at the Department of Surgery, Tokyo Medical University Hospital. The age of patients ranged from 38 to 81 years (average age 63.6 years). There were 87 men and 32 women and cases were pathologically diagnosed as adenocarcinoma in 80 cases, squamous cell carcinomas in 37 cases, adenosquamous carcinoma in one case and large-cell carcinoma in one case. The pathological stage was evaluated according to the TNM classification (Mountain, 1997). The pathological stages of these cases were categorized as: stage IA 28 cases; stage IB 19 cases; stage IIA 6 cases; stage IIB 20 cases; stage IIIA 35 cases; stage IIIB 11 cases.

The surgical materials were fixed by acetone and subsequently embedded in paraffin (Sato et al, 1986). The sections were cut at a thickness of 4 μm, collected on silane-coated slides and stored at 4˚C until use.

### Immunohistochemistry

The immunohistochemical staining was performed with the avidin–biotin peroxidase complex (ABC) method (Hsu et al, 1981) by using a Vectastain ABC Kit. The sections were dewaxed in xylene and rehydrated with graded alcohol. The endogenous peroxidase activity was inhibited by incubation with 0.5% hydrogen peroxidase in methanol for 30 min. The sections were then washed in phosphate-buffered saline (PBS), and incubated in 2% normal swine serum in PBS to block nonspecific binding. The specimens were reacted with each primary antibody overnight at 4˚C. After washing 3 times in PBS, either biotinylated-anti-rabbit, anti-mouse or anti-goat immunoglobulins were used as the second antibody (diluted 1:200). After 30 min of incubation with the second antibody, the sections were washed again with PBS. Avidin–biotin peroxidase complex, diluted 1:200 using a vectastain Kit (Vector Laboratories, Inc Burlingame, CA, USA) was applied for 30 min. After washing, the specimens were reacted with 3,3′-diaminobenzidine tetrahydrochloride (DAB), supplemented with 0.02% hydrogen peroxide in Tris-buffer to visualize the positive area, and then the sections were counterstained with haematoxylin, washed in tap water, dehydrated in alcohol, cleared in xylene and mounted. Negative control slides were prepared using PBS instead of the specific primary antibody.

As the primary antibody, we used anti-VEGF (A-20) rabbit polyclonal antibody (1:400, Santa Cruz Biotechnology, Inc. CA, USA), Anti-bFGF mouse monoclonal antibody (1:200, Wako Pure Chemical Industries, Osaka, Japan), anti-MMP-2 (C-20) goat polyclonal antibody (1:200, Santa Cruz Biotechnology, Inc. CA, USA), and anti-MMP-9 (C-19) goat polyclonal antibody (1:200, Santa Cruz Biotechnology, Inc, CA, USA), anti-c-erbB-2 rabbit polyclonal antibody (Nichirei, Tokyo, Japan), and anti-PD-ECGF mouse monoclonal antibody 1C6-203 (1:200, a gift kindly provided by Dr Tanaka, Nippon Roche Research Center, Kamakura, Japan) (Kono et al, 2001).

### Evaluation of immunohistochemical results

Morphologically histopathological features and immunohistochemical staining were evaluated, independently. More than 300 cancer cells were counted in several fields, which were selected at random. Cases with brown cytoplasm were considered positive. The positive cancer cells were counted and positive rate was calculated. Furthermore, we evaluated the expression levels of each molecule as positive (+: mean – standard deviation or more in positive cells) and negative (–: less than mean – standard deviation in positive cells). We evaluated survival probability according to this classification.

### Statistical analysis

Data were expressed as means ± standard deviation. Statistical analysis was performed using the StatView software system (StatView 5.0.1, SAS Institute Inc). Student's *t*-test was used to analyse the association between categorical clinicopathological variables. For multivariate analysis, logistic regression and stepwise regression analysis were used. Survival curves were calculated from the day of operation by the Kaplan–Meier method and the significance of the difference in the survival rates between the patient groups was calculated by the log-rank test. A $P$ value of less than 0.05 was taken to indicate statistically significant difference.

### RESULTS

### Immunohistochemical analysis of VEGF, bFGF, PD-ECGF, c-erbB-2, MMP-2, MMP-9 based on clinicopathological data

The clinicopathological factors of the 119 patients are summarized in Table 1. Each molecule expressed mainly in the cytoplasm

**Table 1** The clinicopathological factors of the 119 patients with NSCLC

| | Characteristics | No. (%) |
|---|---|---|
| Total | | 119 |
| Sex | Male | 87 (73.1) |
| | Female | 32 (26.9) |
| Age | Mean (years) | 63.6 |
| | Adenocarcinoma | 80 (72.3) |
| Histology | Squamous cell carcinoma | 37 (31.9) |
| | Adenosquamous carcinoma | 1 (0.84) |
| | Large cell carcinoma | 1 (0.84) |
| Tumor size | $T_1$ | 43 (36.1) |
| | $T_2$ | 57 (47.9) |
| | $T_3$ | 12 (10.1) |
| | $T_4$ | 7 (5.9) |
| Lymph node metastasis | – | 56 (47.1) |
| | + | 63 (52.9) |
| Lymphatic permeation | – | 57 (47.9) |
| | + | 53 (44.5) |
| Vascular involvement | – | 58 (48.7) |
| | + | 55 (46.2) |
| Pathological stage | I | 47 (39.5) |
| | II | 26 (21.8) |
| | III | 46 (38.7) |

*© 2001 Cancer Research Campaign* 

of cancer cells. The positive rates of VEGF, bFGF, PD-ECGF, MMP-2, MMP-9 and c-erbB-2 in 119 patients were 76 (63.9%), 92 (77.3%), 68 (57.1%), 87 (73.1%), 78 (65.5%) and 93 (78.2%), respectively. The relationships between expression of these molecules and TNM classification, vascular involvement and lymphatic permeation are shown in Table 2. We divided all patients into one group consisting of pathological T1 (pT1) and pT2, and the other group consisting of pT3 and pT4. There were statistically significant differences in VEGF ($P$ = 0.0001) and MMP-9 ($P$ = 0.001) between the 2 groups (Table 2). 2 pathological N factor (pN-factor) groups were established according to cases with lymph node involvement (N+) and case without lymph node involvement (N–): there were statistically significantly differences in VEGF ($P$ = 0.0019), PD-ECGF ($P$ = 0.034), MMP2 ($P$ = 0.004) and MMP9 ($P$ = 0.009) between the 2 groups. Depending on whether the case is pathological stage I (p-stage I) or not (p stage II, III), statistically significant differences could be observed in VEGF ($P$ = 0.0027) and MMP9 ($P$ = 0.0142) between the 2 p stage groups. Comparing cases according to the presence or absence of either vascular involvement or lymphatic permeation, statistically significant differences were seen in both VEGF ($P$ = 0.0369) and MMP-2 ($P$ = 0.0056) between cases with and cases without lymphatic permeation. However, we did not observe a statistically significant difference in any molecule with regard to vascular involvement.

To investigate which is the most important variable associated with the clinicopathological factors, stepwise regression and logistic regression analysis were performed. As a result of multivariate analysis, we found VEGF was the most closely associated with local invasion (pT1 and pT2 vs. pT3 and pT4: $P$ = 0.0084), and MMP-2 was the only characteristic to have a significant independent impact on lymphatic permeation ($P$ = 0.0228).

### Relationship between expression levels of each molecule and prognosis

For survival analyses, survival data were available for 111 patients. Patients dying within 60 days of surgery were excluded to avoid bias from perioperative death. The deaths of all other patients were cancer-related death. The 3-year and 5-year survival rates for the 111 patients were 59.4% and 47.4%, respectively, with a median survival of 36.0 months. Univariate analysis of survival probability showed that the expression of VEGF ($P$ = 0.0083), bFGF ($P$ = 0.0173), MMP-2 ($P$ = 0.0149), MMP-9 ($P$ = 0.0126) were significantly associated with a worse prognosis (Figure 1A–D). The 5-year survival rates of cases with VEGF, bFGF, MMP-2 and MMP-9 positive were 37.5%, 41.2%, 40.9%, 38.4%, respectively, whereas they were 64.4%, 73.6%, 69.0% and 68.3% when VEGF, bFGF, MMP-2 and MMP-9 were negative. No association was observed between expression of PD-ECGF ($P$ = 0.8198) or c-erbB-2 ($P$ = 0.3004) and survival probability (Figure 1E, F).

Cases with co-expression of VEGF and MMP-2 were found in 65 out of the 111 (58.6%) and were associated with a poor

**Table 2** Relationships between expression of VEGF, bFGF, PD-ECGF, c-erbB-2, MMP-2, MMP-9 and clinicopathological factors in 119 patients with NSCLC

| | No. of cases | Expression (mean ± SD) (%) | | | | | |
|---|---|---|---|---|---|---|---|
| | | VEGF | bFGF | PD-ECGF | MMP-2 | MMP-9 | c-erbB-2 |
| pT-factor | | | | | | | |
| PT1, pT2 | 43 | 25.58 ± 27.63 | 39.30 ± 24.04 | 26.28 ± 26.64 | 36.74 ± 26.34 | 24.19 ± 19.18 | 49.07 ± 30.22 |
| PT3, pT4 | 76 | 45.40 ± 25.16 | 48.16 ± 24.64 | 35.92 ± 26.34 | 43.82 ± 24.38 | 37.24 ± 21.33 | 50.40 ± 28.21 |
| *P* value | | 0.0001 | 0.0599 | 0.0585 | 0.1426 | 0.0012 | 0.8109 |
| pN-factor | | | | | | | |
| PN (–) | 56 | 30.00 ± 26.22 | 40.36 ± 27.43 | 26.96 ± 25.36 | 34.29 ± 25.36 | 27.14 ± 20.25 | 48.75 ± 31.51 |
| PN (+) | 63 | 45.56 ± 27.05 | 49.05 ± 21.38 | 37.30 ± 27.19 | 47.46 ± 23.62 | 37.30 ± 21.49 | 50.95 ± 26.44 |
| *P* value | | 0.0019 | 0.0550 | 0.0347 | 0.0040 | 0.0093 | 0.6793 |
| p stage | | | | | | | |
| I | 47 | 28.94 ± 25.73 | 39.57 ± 26.94 | 28.30 ± 25.73 | 35.75 ± 26.19 | 25.60 ± 20.88 | 50.43 ± 31.55 |
| II, III | 72 | 44.31 ± 27.36 | 48.47 ± 22.62 | 35.14 ± 27.22 | 44.86 ± 24.09 | 36.39 ± 21.05 | 49.58 ± 27.14 |
| *P* value | | 0.0027 | 0.0543 | 0.1736 | 0.0536 | 0.0142 | 0.8770 |
| Vascular involvement[a] | | | | | | | |
| (–) | 58 | 35.35 ± 28.73 | 43.79 ± 24.63 | 31.38 ± 25.44 | 37.93 ± 26.07 | 29.83 ± 21.23 | 51.90 ± 29.23 |
| (+) | 55 | 40.36 ± 25.89 | 46.73 ± 25.10 | 30.91 ± 25.84 | 45.27 ± 24.86 | 36.00 ± 21.31 | 47.82 ± 29.36 |
| *P* value | | 0.3323 | 0.5318 | 0.9225 | 0.1288 | 0.1260 | 0.4610 |
| Lymphatic permeation[b] | | | | | | | |
| (–) | 57 | 32.98 ± 26.12 | 44.56 ± 24.35 | 32.81 ± 26.91 | 35.26 ± 24.50 | 30.18 ± 21.92 | 45.79 ± 27.90 |
| (+) | 53 | 43.77 ± 28.03 | 46.42 ± 24.42 | 31.32 ± 25.27 | 48.68 ± 25.27 | 36.60 ± 21.39 | 53.96 ± 29.70 |
| *P* value | | 0.0389 | 0.6912 | 0.7662 | 0.0056 | 0.1229 | 0.1396 |

[a]6 patients can not be confirmed.[b]9 patients can not be confirmed.

 *© 2001 Cancer Research Campaign*

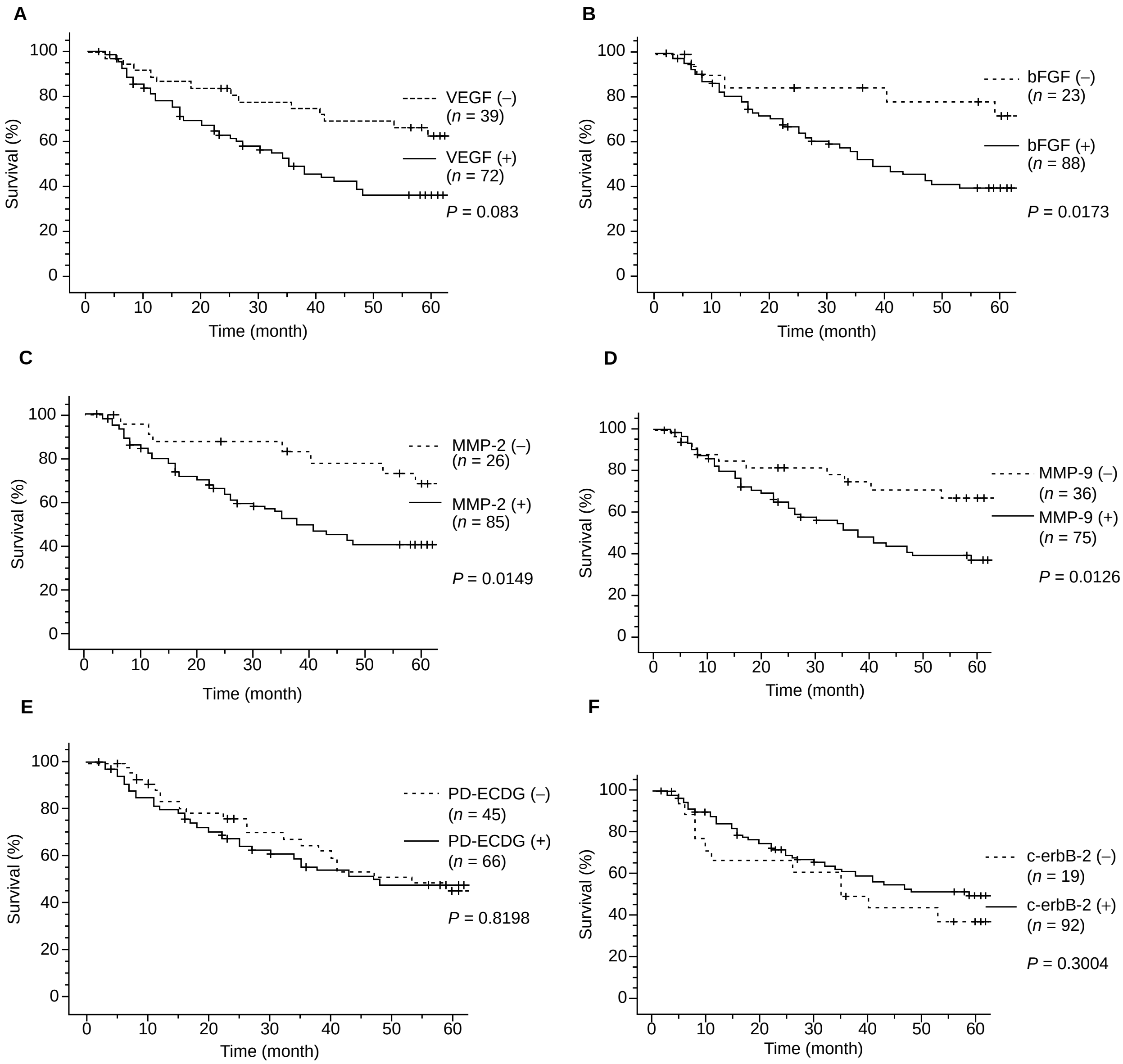

**Figure 1** Kaplan–Meier survival curves for 111 patients with NSCLC. *P* value was determined with the log-rank test. (**A**) VEGF (–) vs. VEGF (+). The 5-year survival rate was 37.5% in VEGF-positive cases, whereas it was 64.4% in VEGF-negative cases (*P* = 0.0083). (**B**) bFGF (–) vs. bFGF (+). The 5-year survival rate was 41.2% in bFGF-positive cases, whereas it was 64.4% in bFGF-negative cases (*P* = 0.000.0173). (**C**) MMP- 2 (–) vs. MMP-2 (+). The 5-year survival rate was 40.9% in MMP-2-positive cases, whereas it was 69.0% in MMP-2-negative cases (*P* = 0.0.0149). (**D**) MMP-9 (–) vs. MMP-9 (+). The 5-year survival rate was 38.4% in MMP-9-positive cases, but it was 68.3% in MMP-9-negative cases (*P* = 0.0126). (**E**) PD-ECGF (–) vs. PD-ECGF (+). The 5-year survival rate was 46.5% in PD-ECGF-positive cases, and it was 45.9% in PD-ECGF-negative cases (*P* = 0.8198). (**F**) c-erbB-2 (–) vs. c-erbB-2 (+). The 5-year survival rate was 49.6% in c-erbB-2-positive cases, and it was 37.5% in c-erbB-2-negative cases (*P* = 0.3004)

outcome (*P* = 0.0008, Figure 2). The 5-year survival rate in cases with co-expression of VEGF and MMP-2 was only 32.9%, but it was 66.7% when the expressions of both VEGF and MMP-2 or one of them were negative.

### DISCUSSION

The processes of cancer cell invasion to adjacent tissues and distant metastasis consist of a complex series of sequential step. Therefore, we can speculate that several molecular mechanisms involving specific cancer cells and host characteristics are probably deeply involved. Angiogenesis seems to be one of the most important processes of cancer progression, and consists of proteolysis of the extracellular matrix, proliferation and migration of endothelial cells, as well as the synthesis of new matrix components. In general, it is known that primary lung cancer is one of the most malignant solid tumours, and that its potential for local invasion and distant metastasis is great. In the present study we attempted to investigate the immunohistochemical expression of VEGF, bFGF, PD-ECGF, MMP-2, MMP-9 and c-erbB-2, which are associated with angiogenesis and proliferation, using surgically resected specimens of NSCLC and to evaluate the

© 2001 Cancer Research Campaign

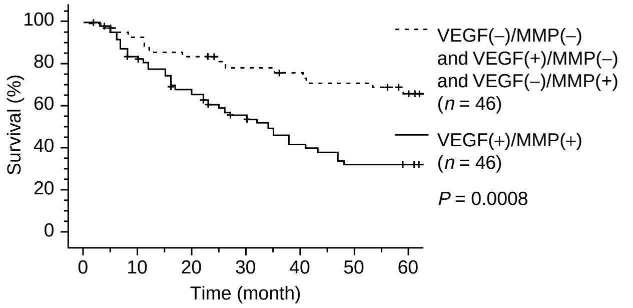

**Figure 2** Kaplan–Meier survival curves for 111 patients with NSCLC. *P* value was determined with the log-rank test. The 5-year survival rate was 32.9% in cases with co-expression of VEGF and MMP-2, whereas it was 66.7% in cases with either or both VEGF and MMP-2 negative. Co-expression of VEGF and MMP-2 was significantly associated with a poor outcome ($P = 0.0008$)

relationship between the expression levels of each molecule and clinicopathological factors or prognosis.

### VEGF

Several previous studies have showed that angiogenesis play an important role in the cell growth, progression and metastasis of solid tumours. Ohta et al (1997, 1999) reported a significant correlation between VEGF expression and both tumour size and lymph node metastasis in primary lung cancer, and they recognized that VEGF expression was associated not only with distant metastasis but also with lymphatic metastasis. Some investigators also have found a significant correlation between VEGF expression and poor prognosis in NSCLC (Fontanini et al, 1997; Giatromanolaki et al, 1998; Oshika et al 1998; Volm et al, 1999; Ohta et al, 1999). These data support our present results, that we found the VEGF expression levels were significantly associated with several kinds of clinicopathological factors and prognosis.

### bFGF

There are several reports concerning the relationship between bFGF expression and the incidence of metastasis. Though statistically significant differences could not be recognized in pancreatic carcinoma (Ohta et al, 1995), bFGF seems to be a valuable marker for lymph node metastasis or distant metastasis in renal cell carcinoma and gastric carcinoma (Duensing et al, 1995; Ueki et al, 1995; Volm et al, 1999). Several studies concerning NSCLC showed that bFGF expression was related to progression and prognosis (Takanami et al, 1996; Volm et al, 1997). However, according to another investigation, bFGF-immunoreactivity of cancerous cells did not correlate with histological-type tumour size, nodal status and stage in NSCLC, even though bFGF-immunoreactivities of stromal cells were associated with lymph node metastasis and advanced pathological stage in NSCLC (Guddo et al, 1999). In our own results, no statistically significant relationship could be observed between bFGF expression levels and any clinicopathological factor. However, we found there is a significant association between-bFGF expression and prognosis.

### PD-ECGF

PD-ECGF is identical to thymidine phosphorylase and possesses angiogenic activity (Moghaddam and Bicknell, 1992). In colorectal carcinoma, high expression of PD-ECGF was related to the extent of tumour invasion, lymphatic and vascular involvement (Takebayashi et al, 1996). However, another investigator reported that it was not associated with histological type, the depth of tumour invasion or lymph node involvement (Maeda et al, 1995). There is a report concerning NSCLC, in which PD-ECGF did not prove to be a significantly prognostic factor, but a statistically significant correlation of PD-ECGF expression with the pT-factor was recognized (Koukourakis et al, 1997). Our data partially support those previous results, and show a weak relationship without statistical significance between PD-ECGF and the pT-factor ($P = 0.059$). Furthermore, although we recognized a statistically significant difference of PD-ECGF expression in relation to the pN-factor ($P = 0.0347$), no association between PD-ECGF expression and prognosis could be observed.

### c-erbB-2

This molecule is well known as a prognostic factor for breast cancer and ovarian cancer (Slamon et al, 1987; Tsuda et al, 1989; Hengstler et al, 1999), and is deeply associated with cell growth (Giani et al, 1998). According to previous reports concerning primary lung cancer, the range of overexpression rate is wide (20–80%) (Kern et al, 1990; Tateishi et al, 1991). Most reports revealed no statistically significant difference between the overexpression and survival probability in adenocarcinoma of the lung (Giatromanolaki et al, 1996; Pfeiffer et al, 1996). In our study the positive rate of c-erbB-2 was 78.2%, and no statistically significant difference could be recognized concerning clinicopathological factors and prognosis.

### MMP

The MMPs, which belong to extracellular endopeptidases, selectively degrade components of the extracellular matrix. Recent studies showed that the activity of several kinds of MMPs had increased in the early stages of tumour progression. Especially, the activities of MMP-2 and MMP-9 were remarkably increased (Aznavoorian et al, 1993; Wilson and Matrisian, 1996). Overexpression of MMPs was associated with local invasion to the adjacent tissues or distant metastasis in NSCLC (Gonzalez-Avila et al, 1998; Suzuki et al, 1998). MMP-2 has been implicated in lymphatic and vascular invasion of NSCLC (Brown et al, 1993). More recently, the MMPs themselves are considered to be angiogenesis-associated proteins, because either synthetic or endogenous MMP inhibitors inhibit angiogenetic reactivity (Hiraoka et al, 1998). Another study provides direct evidence that MMP-2-deficient mice exhibit the reduction of angiogenetic response and tumour progression in tumour xenografts (Itoh et al, 1998). Moreover, though capillary endothelial cells cultured on 2-dimensional type I collagen gels produced low levels of pro-MMP-2 with little endogenous activation, in 3 dimensional type I collagen gels there was a marked increase in pro-MMP-2, and endothelial cells organize lumen formation in multicellular structures (Haas et al, 1998). Clinically, many investigators reported the prognostic value of MMP-2 and MMP-9 in NSCLC (Kodate et al, 1997; Cox et al, 2000; Passlick et al, 2000). In our study, MMP-2 positivity was associated with the pN-factor and lymphatic permeation. MMP-9 showed a significant correlation to pT-factor, pN-factor and p-stage. There were significant relationships between MMP-2, MMP-9 expression and survival probability.

Based on multivariate analysis we found VEGF and MMP-2 were independent characteristics affecting the pT-factor and

 *© 2001 Cancer Research Campaign*

lymphatic permeation, respectively. Garzetti et al (1999) reported that there was a significant relationship between VEGF and MMP-2 in immunohistochemical analysis of serous ovarian tumours, and that the VEGF-positive group showed worse disease-free survival than the VEGF-negative group. However, there has been no report concerning the co-expression of VEGF and MMP-2 in NSCLC. Recently, it was reported that MMPs-2 and -9 are up-regulated in angiogenic lesions and that MMP-9 could render pancreatic islets in transgenic mice angiogenic, releasing VEGF (Bergers et al, 2000). It may be suggested that there are some interactions at the molecular level between MMPs and VEGF. We found co-overexpression of VEGF and MMP-2 in 65 out of 111 cases (58.6%) and this was significantly associated with a poor outcome. Our present investigation suggested that VEGF and MMP-2 might possess the most valuable prognostic impact. Therefore, our data support the hypothesis that MMPs might generate bio-available VEGF.

Based on the evidence that angiogenesis plays a crucial role in tumour progression, angiogenetic factors and proteases are novel targets for chemotherapeutic strategy, and the use of these inhibitors requires further evaluation. MMP inhibitors demonstrated efficacy in preclinical studies using experimental animals and model system (Johnson et al, 1998; Belotti et al, 1999; Curran and Murray, 1999; Nelson et al, 2000; Ohta et al, 2001). However, no clinical efficacy was demonstrated in phrase III drug trials of MMP inhibitors (Zucker et al, 2000). In spite of considerable recent progress in identifying the multi-functions of MMPs, MMP inhibitors and VEGF in cancerous lesions, understanding of the mechanisms of tumour progression is far from complete. The present types of clinicopathological investigation may provide a basis not only for evaluation of tumour malignancy but also for the preselection of patients to be included in clinical trials to investigate the benefit of new kinds of adjuvant therapy.

### ACKNOWLEDGEMENTS

We would like to thank Professor J Patrick Barron and T Kojima of the International Medical Communications Center at Tokyo Medical University for reviewing the manuscript. We are grateful to Nippon Roche Research Center for the gift of anti-PD-ECGF antibody. This study was supported in part by the Japan-China Sasakawa Medical Fellowship.

### REFERENCES

Aznavoorian S, Murphy AN, Stetler-Stevenson WG and Liotta LA (1993) Molecular aspects of tumor cell invasion and metastasis. *Cancer* **71**: 1368–1383

Brown PD, Bloxidge RE, Stuart NS, Gatter KC and Carmichael J (1993) Association between expression of activated 72-kilodalton gelatinase and tumor spread in non-small-cell lung carcinoma. *J Natl Cancer Inst* **85**: 574–578

Belotti D, Paganoni P and Giavazzi R (1999) MMP inhibitors: experimental and clinical studies. *Int J Biol Markers* **14**: 232–238

Bergers G, Brekken R, McMahon G, Vu TH, Ito T, Tamaki K, Tanzawa K, Thorpe P, Itohata S, Werb Z and Hanahan D (2000) Matrix metalloproteinase– 9 triggers the angiogenic switch during carcinogenesis. *Nat Cell Biol* **2**: 737–744

Cox G, Jones JL and O'Byrne KJ (2000) Matrix metalloproteinase-9 and the epidermal growth factor signal pathway in operable non-small cell lung cancer. *Clin Cancer Res* **6**: 2349–2355

Curran S and Murray GI (1999) Matrix metalloproteinases in tumour invasion and metastasis. *J Pathol* **189**: 300–308

Duensing S, Grosse J and Atzpodien J (1995) Increased serum levels of basic fibroblast growth factor (bFGF) are associated with progressive lung metastases in advanced renal cell carcinoma patients. *Anticancer Res* **15**: 2331–2333

el-Torky M, el-Zeky F and Hall JC (1990) Significant changes in the distribution of histologic types of lung cancer. A review of 4928 cases. *Cancer* **65**: 2361–2367

Folkman J (1985) Tumor angiogenesis. *Adv Cancer Res* **43**: 175–203

Fontanini G, Vignati S, Boldrini L, Chine S, Silvestri V, Lucchi M, Mussi A, Angeletti CA and Bevilacqua G (1997) Vascular endothelial growth factor is associated with neovascularization and influences progression of non-small cell lung carcinoma. *Clin Cancer Res* **6**: 861–865

Fujise N, Nanashim A, Taniguchi Y, Matsuo S, Hatano K, Matsumoto Y, Tagawa Y and Ayabe H (2000) Prognostic impact of cathepsin B and matrix metalloproteinase-9 in pulmonary adenocarcinomas by immunohistochemical study. *Lung Cancer* **27**: 19–26

Garzetti GG, Ciavattini A, Lucarini G, Pugnaloni A, De Nictolis M, Amati S, Romanini C and Biagini G (1999) Expression of vascular endothelial growth factor related to 72-kilodalton metalloproteinase immunostaining in patients with serous ovarian tumors. *Cancer* **85**: 2219–2225

Gasparini G (1996) Angiogenesis research up to 1996. A commentary on the state of art and suggestions for further studies. *Eur J Cancer* **32A**: 2379–2385

Giani C, Casalini P, Pupa SM, De Vecchi R, Ardini E, Colnaghi MI, Giordano A and Menard S (1998) Increased expression of c-erbB-2 in hormone-dependent breast cancer cells inhibits cell growth and induces differentiation. *Oncogene* **17**: 425–432

Giatromanolaki A, Gorgoulis V, Chetty R, Koukourakis MI, Whitehouse R, Kittas C, Veslemes M, Gatter KC and Iordanoglou I (1996) C-erbB-2 oncoprotein expression in operable non-small cell lung cancer. *Anticancer Research* **16**: 987–993

Giatromanolaki A, Koukourakis MI, Kakolyris S, Turley H, O'Byrne K, Scott PA, Pezzella F, Georgoulias V, Harris AL and Gatter KC (1998) Vascular endothelial growth factor, wild-type p53, and angiogenesis in early operable non-small cell lung cancer. *Clin Cancer Res* **4**: 3017–3024

Ginsberg RJ and Rubinstein LV (1995) Randomized trial of lobectomy versus limited resection for T1 No non-small cell lung cancer. Lung Cancer Study Group. *Ann Thorac Surg* **60**: 615–22; discussion 622–623

Gonzalez-Avila G, Iturria C, Vadillo F, Teran L, Selman M and Perez-Tamayo R (1998) 72-kD (MMP-2) and 92-kD (MMP-9) type IV collagenase production and activity in different histologic types of lung cancer cells. *Pathobiology* **66**: 5–16

Guddo F, Fontanini G, Reina C, Vignola AM, Angeletti A and Bonsignore G (1999) The expression of basic fibroblast growth factor (bFGF) in tumor-associated stromal cells and vessels is inversely correlated with non-small cell lung cancer progression. *Hum Pathol* **30**: 788–794

Haas TL, Davis SJ and Madri JA (1998) Three-dimensional type-I collagen lattices induce coordinate expression of matrix metalloproteinases MT1-MMP and MMP-2 in microvascular endothelial cells. *J Biol Chem* **273**: 3604–3610

Hengstler JG, Lange J, Kett A, Dornhofer N, Meinert R, Arand M, Knapstein PG, Becker R, Oesch F and Tanner B (1999) Contribution of c-erbB-2 and topoisomerase IIalpha to chemoresistance in ovarian cancer. *Cancer Res* **59**: 3206–3214

Hiraoka N, Allen E, Apel IJ, Gyetko MR and Weiss SJ (1998) Matrix Metalloproteinase regulate neovascularization by acting as pericellular fibrinolysins. *Cell* **95**: 365–377

Hsu SM, Raine L and Fanger H (1981) Use of avidin-biotin-peroxidase complex (ABC) in immunoperoxidase techniques: a comparison between ABC and unlabeled antibody (PAP) procedures. *J Histochem Cytochem* **29**: 577–580

Ito T, Tanioka M, Yoshida H, Yoshioka T, Nishimoto H and Itohara S (1998) Reduced angiogenesis and tumor progression in gelatinase A-deficient mice. *Cancer Res* **58**: 1048–1051

Johnson LJ, Dyer R and Hupe DJ (1998) Matrix metalloproteinases. *Curr Opin Chem Biol* **2**: 466–471

Kern JA, Schwartz DA, Nordberg JE, Weiner DB, Greene MI, Torney L and Robinson RA (1990) p185neu expression in human lung adenocarcinomas predicts shortened survival. *Cancer Res* **50**: 5184–5187

Kleiner DE and Steler-stevenson WG (1999) Matrix metalloproteinases and metastasis. *Cancer Chemther Pharmacol* **43** (**Suppl**): S42–S51

Kodate M, Kasai T, Hashimoto H, Yasumoto K, Iwata Y and Manabe H (1997) Expression of matrix metalloproteinase (gelatinase) in T1 adenocarcinoma of the lung. *Pathol Int* **47**: 461–469

Kono T, Nishida M, Inagaki N, Tanaka Y, Yoneda M and Kasai S (2001) Development and characterization of 1C6-203, a new monoclonal antibody specific to human thymidine phosphorylase. *J Histochem Cytochem* **49**: 131–138

Koukourakis MI, Giatromanolaki A, O'Byrne KJ, Comley M, Whitehouse RM, Talbot DC, Gatter KC and Harris AL (1997) Platelet-derived endothelial cell growth factor expression correlates with tumour angiogenesis and prognosis in non-small-cell lung cancer. *Br J Cancer* **75**: 477–481

© 2001 Cancer Research Campaign 

Maeda K, Chung YS, Ogawa Y, Takatsuka S, Sawada T, Onoda N, Nitta A, Arimoto Y and Sowa M (1995) Malignancy of gastric cancer analyzed by the expression of thymidine phosphorylase. *Gan To Kagaku Ryoho* **22**: 679–682 (Japanese)

Moghaddam A and Bicknell R (1992) Expression of platelet-derived endothelial cell growth factor in Escherichia coli and confirmation of its thymidine phosphorylase activity. *Biochemistry* **31**: 12141–12146

Mountain CF (1997) Revisions in the International System for Staging Lung Cancer. *Chest* **111**: 1710–1717

Nelson AR, Fingleton B, Rothenberg ML and Matrisian LM (2000) Matrix metalloproteinses: biological activity and clinical implications. *J Clin Oncol* **18**: 1135–1149

O'Byrne KJ, Koukourakis MI, Giatromanolaki A, Cox G, Turley H, Steward WP, Gatter K and Harris AL (2000) Vascular endothelial growth factor, platelet-derived endothelial cell growth factor and angiogenesis in non-small-cell lung cancer. *Br J Cancer* **82**: 1427–1432

Ohta M, Konno H, Tanaka T, Baba M, Kamiya K, Oba Kouji, Kaneko T, Syouji T, Igarashi A and Nakamura S (2001) Effect of combination therapy with matrix metalloproteinase inhibitor MMI-166 and Mitomycin C on the growth and liver metastasis of human colon cancer. (Japan) *J Cancer Res* **92**: 688–695

Ohta T, Yamamoto M, Numata M, Iseki S, Tsukioka Y, Miyashita T, Kayahara M, Nagakawa T, Miyazaki I, Nishikawa K and Yoshitake Y (1995) Expression of basic fibroblast growth factor and its receptor in human pancreatic carcinomas. *Br J Cancer* **72**: 824–831

Ohta Y, Watanabe Y, Murakami S, Oda M, Hayashi Y and Nonomura A (1997) Vascular endothelial growth factor and lymph node metastasis in primary lung cancer. *Br J Cancer* **76**: 1041–1045

Ohta Y, Tomita Y, Oda M, Watanabe S, Murakami S and Watanabe Y (1999) Tumour angiogenesis and recurrence in stage I non-small cell lung cancer. *Ann Thorac Surg* **68**: 1034–1038

Oshika Y, Nakamura M, Tokunaga T, Ozeki Y, Fukushima Y, Hatanaka H, Abe Y, Yamazaki H, Kijima H, Tamaoki N and Ueyama Y (1998) Expression of cell-associated isoform of vascular endothelial growth factor 189 and its prognostic relevance in non-small cell lung cancer. *Int J Oncol* **12**: 541–544

Passlick B, Sienel W, Seen-Hibler R, Wockel W, Thetter O, Mutschler W and Pantel K (2000) Overexpression of matrix metalloproteinase 2 predicts unfavorable outcome in early-stage non-small cell lung cancer. *Clin Cancer Res* **6**: 3944–3948

Pfeiffer P, Clausen PP, Andersen K and Rose C (1996) Lack of prognostic significance of epidermal growth factor receptor and the oncoprotein p185HER-2 in patients with systemically untreated non-small-cell lung cancer: an immunohistochemical study on cryosections. *Br J Cancer* **74**: 86–91

Sato Y, Mukai K, Watanabe S, Goto M and Shimosato Y (1986) The AMeX method. A simplified technique of tissue processing and paraffin embedding with improved preservation of antigens for immunostaining. *Am J Pathol* **125**: 431–435

Slamon DJ, Clark GM, Wong SG, Levin WJ, Ullrich A and McGuire WL (1987) Human breast cancer: correlation of relapse and survival with amplification of the HER-2/neu oncogene. *Science* **235**: 177–182

Stetler-Stevenson WG, Hewitt R and Corcoran M (1996) Matrix metalloproteinases and tumor invasion: from correlation and causality to the clinic. *Semin Cancer Biol* **7**: 147–154

Suzuki M, Iizawa T, Fujisawa T, Baba M, Yamaguchi Y, Kimura H and Suzuki H (1998) Expression of matrix metalloproteinases and tissue inhibitor of matrix metalloproteinases in non-small cell lung cancer. *Invasion metastasis* **18**: 134–141

Takanami I, Tanaka F, Hashizume T, Kikuchi K, Yamamoto Y, Yamamoto T and Kodaira S (1996) The basic fibroblast growth factor and its receptor in pulmonary adenocarcinomas: an investigation of their expression as prognostic markers. *Eur J Cancer* **32A**: 1504–1509

Takebayashi Y, Akiyama S, Akiba S, Yamada K, Miyadera K, Sumizawa T, Yamada Y, Murata F and Aikou T (1996) Clinicopathologic and prognostic significance of an angiogenic factor, thymidine phosphorylase, in human colorectal carcinoma. *J Natl Cancer Inst* **88**: 1110–1117

Tateishi M, Ishida T, Mitsudomi T, Kaneko S and Sugimachi K (1991) Prognostic value of c-erbB-2 protein expression in human lung adenocarcinoma and squamous cell carcinoma. *Eur J Cancer* **27**: 1372–1375

Tsuda H, Hirohashi S, Shimosato Y, Hirota T, Tsugane S, Yamamoto H, Miyajima N, Toyoshima K, Yamamoto T, Yokota J et al (1989) Correlation between long-term survival in breast cancer patients and amplification of two putative oncogene- coamplification units: hst-1/int-2 and c-erbB-2/ear-1. *Cancer Res* **49**: 3104–3108

Ueki T, Koji T, Tamiya S, Nakane PK and Tsuneyoshi M (1995) Expression of basic fibroblast growth factor and fibroblast growth factor receptor in advanced gastric carcinoma. *J Pathol* **177**: 353–361

Volm M, Koomagi R, Mattern J and Stammler G (1997) Angiogenic growth factors and their receptors in non-small cell lung carcinomas and their relationships to drug response in vitro. *Anticancer Res* **17**: 99–103

Volm M, Koomagi R and Mattern J (1999) PD-ECGF, bFGF, and VEGF expression in non-small cell lung carcinomas and their association with lymph node metastasis. *Anticancer Res* **19**: 651–655

Wilson CL and Matrisian LM (1996) Matrilysin: an epithelial matrix metalloproteinase with potentially novel functions. *Int J Biochem Cell Biol* **28**: 123–136

Yano T, Tanikawa S, Fujie T, Masutani M and Horie T (2000) Vascular endothelial growth factor expression and neovascularisation in non-small cell lung cancer. *Eur J Cancer* **36**: 601–609

Zucker S, Cao J and Chen W (2000) Critical appraisal of the use of matrix metalloproteinase inhibitors in cancer treatment. *Oncogen* **19**: 6642–6650

*© 2001 Cancer Research Campaign*
